# Supplementary material for: Surface-Modified Inhaled Microparticle-Encapsulated Celastrol for Enhanced Efficacy in Malignant Pleural Mesothelioma
Source: Int J Mol Sci. 2023 Mar 8;24(6):5204. doi: 10.3390/ijms24065204 (PMC10049545; doi:10.3390/ijms24065204)
Supplement: Supplementary file 1 [file ijms-24-05204-s001.zip › ijms-2192168-supplementary.pdf]

Supplementary Information

Surface-Modified Inhaled Microparticle-encapsulated Celastrol for Enhanced Efficacy in Malignant Pleural Mesothelioma

Xuechun Wang[a], Gautam Chauhan[a], Alison RL Tacderas[b], Aaron Muth[a], Vivek Gupta[a,*]

Methods

Cytotoxicity Studies

Mesothelioma cells were seeded in tissue-culture (TC) treated 96-well plates (Eppendorf, Hauppauge, NY, USA) at a cell density of 2,500 cells/well. After overnight incubation, treatments were added at varying Cela concentrations ranging from 0.10 to 6.25 µM. Equivalent volumes of Cela MP were chosen based on drug entrapment. Untreated cells were designated as the control. After 48 h incubation, treatments were replaced with the MTT solution (1 mg/mL in sterile 1x PBS) and incubated for 2 h, then replaced with dimethyl sulfoxide (DMSO), followed by shaking for 30 min. The amount of formazan was determined by measuring the absorbance at 570 nm using a Spark 10M Plate Reader (Tecan, Männedorf, Switzerland).

DPPH Antioxidant Assay

First, a stock solution of DPPH (0.2 mM) in methanol was prepared. MPs were diluted in methanol (1 mL) and added to the DPPH stock to obtain a final volume of 2 mL with 0.1 mM DPPH (sample). The control was prepared by mixing 1 mL of DPPH stock with 1 mL of milliQ water (control). The blank was comprised of 1 mL sample diluted in methanol combined with 1 mL methanol (blank). Absorbance readings (A_{sample}, $A_{control}$, A_{blank}) were taken after 0.5 and 24 h at 518 nm using a UV 1600 PC spectrophotometer (VWR, Radnor, PA, USA). **Equation S1** shows the calculation for DPPH scavenging effect (%):

$$DPPH\ Scavenging\ Effect\ (\%) = \left(1 - \frac{A_{sample} - A_{blank}}{A_{control}}\right) \times 100\% \quad \ldots\ldots \boldsymbol{Eq.S1}$$

Effect of Cela and Cela MP on Cellular Autophagy

MSTO-211H cells were seeded in 96-well plates (25,000 cells/well) and were incubated with serum-free media to induce a starvation-linked autophagy process for 24 h. Starved cells were then treated with Cela or Cela MP (5.0 µM) for an additional 18 h as per the manufacturer's protocol. Treatments were then replaced with 1X assay buffer (100 µL) followed by the addition of a dual color detection reagent and incubation for 30 min in the dark at 37 °C (100 µL of CYTO-ID® Green Detection Reagent + Hoechst 33,342 nuclear stain growth medium without phenol red indicator supplemented with 5% FBS). After incubation, excess dye was removed by washing with 1X assay buffer and 100 µL of 1X assay buffer was added to each well. CYTO-ID® Green Detection Reagent was read with a FITC filter (480 nm/530 nm ex/em), and the Hoechst 33,342 Nuclear Stain was read with a DAPI filter set (340 nm/480 nm ex/em) using a Spark 10M plate reader.

Results

UPLC Method Development of Cela

Short elution time and intense, indicative peak were accomplished using the UPLC method for analyzing Cela. The mobile phase composition containing 0.1% OPA: ACN at ratio of 10:90 with 0.8 mL/min flow rate was found to be most acceptable. Further, a strong linear calibration curve was plotted ranging from 1-100 µg/mL. As shown in **Figure S1**, a good symmetric peak of Cela with the retention time at 1.226 min was observed.

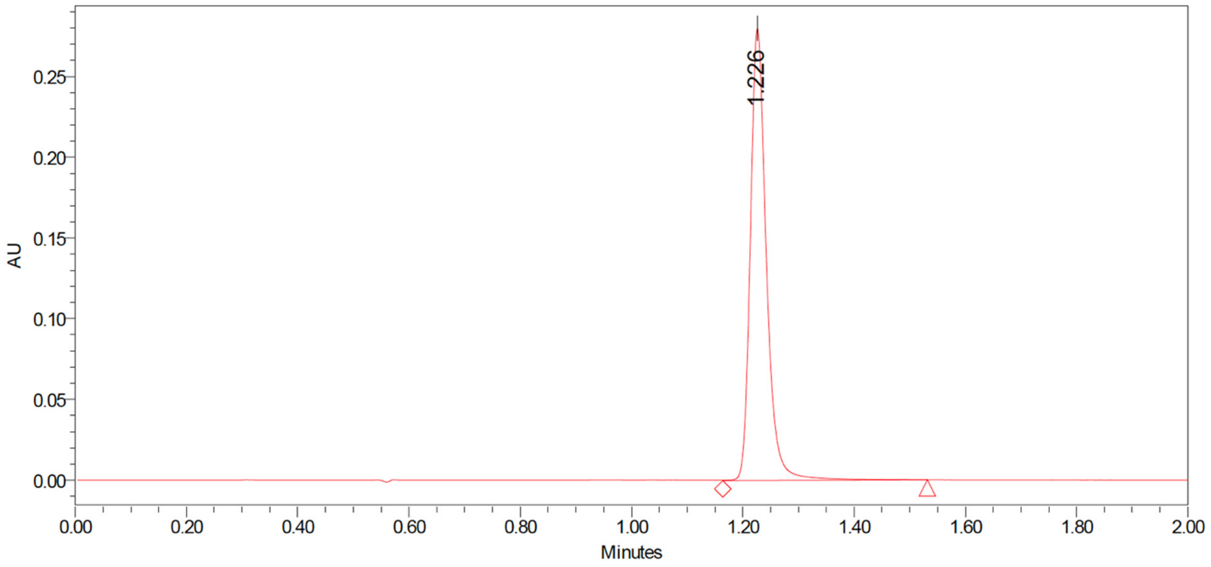

Figure. S1: Representative UPLC chromatogram of Celastrol (Cela)

Table S1: Methods and physical characterizations for formulations F1-F5 of Cela MP formulation optimization.

No.	A1	O1	A2	% Entrapment Efficiency	Particle Size (nm)	PDI	Zeta Potential (mV)
F1	Water	2 mg drug in DCM and DMSO	1% PVA	63.7	831.1	0.114	-30.3
F2	Water + 1% PEI	2 mg drug in DCM and DMSO	1% PVA	60.3	1,152	0.518	39.6
F3	Water	2 mg drug in DCM and DMSO	2% PVA	67.1	886.7	0.185	-32.9
F4	Water + 1% PEI	2mg drug in DCM and DMSO	2% PVA	52.0	1,078	0.033	35.7
F5	Water + 1% $NaHCO_3$	2mg drug in DCM and DMSO	1% PVA	34.8	2,341	0.791	-21.6

A1 – Inner aqueous phase
O1 – Organic phase
A2 – Outer aqueous phase

Table S2: Physical characterizations of F6 Cela MPs after nebulization.

% Entrapment Efficiency	Particle Size (nm)	PDI	Zeta Potential (mV)
68.1±3.4	2,098±89	0.1±0.1	-35.2±1.1
